# A 25-year evaluation of pharyngeal airways dimensional changes after headgear orthopedic treatment

**DOI:** 10.1590/2177-6709.30.4.e252552.oar

**Published:** 2025-11-07

**Authors:** Taís de Morais Alves da CUNHA, Telma Martins de ARAÚJO, Carlos Jorge VOGEL, Emanuel Braga RÊGO, Eustáquio Afonso ARAÚJO, Ki Beom KIM

**Affiliations:** 1 Saint Louis University, School of Dentistry, Department of Orthodontics (Missouri, USA).; 2 Universidade Federal da Bahia, Faculdade de Odontologia, Departamento de Ortodontia (Salvador/BA, Brazil).

**Keywords:** Cephalometry, Nasopharynx, Extraoral traction appliances, Malocclusion, Angle Class II, Cefalometria, Nasofaringe, Aparelhos de tração extrabucal, Má oclusão Classe II de Angle

## Abstract

**Introduction:**

Nasopharyngeal obstruction may influence craniofacial growth, lead to psychosocial impairments, and adversely affect quality of life. Thus, it is important to identify the potential of orthodontic therapies to alter the dimensions of the airways.

**Objectives:**

To evaluate the long-term changes in pharyngeal airway dimensions in skeletal Class II patients treated with cervical headgear appliance.

**Material and Methods:**

A retrospective study was conducted with 20 patients treated by an experienced Orthodontist. The airway space was assessed using linear and area measurements on cephalometric radiographs before treatment, after treatment, and at a 25-year follow-up.

**Results:**

measurements showed high to moderate reliability. None of the measures showed a decrease after treatment. The nasopharyngeal, oropharyngeal, and hypopharyngeal areas were not modified after headgear intervention and showed a tendency to narrow over the follow-up period. Regarding linear measurements, the nasopharyngeal dimensions remained unchanged or increased after treatment, returning to initial dimensions in the follow-up assessment.

**Conclusion:**

Orthopedic treatment with extraoral appliances did not cause a decrease in pharyngeal airway dimension and is considered safe for the treatment of growing skeletal Class II malocclusions according to the results of the present study.

## RESUMO

**Introdução:**

A obstrução nasofaríngea pode alterar o crescimento craniofacial além de provocar impacto psicossocial e na qualidade de vida. Dessa forma é importante identificar o potencial de terapias ortodônticas em alterar as dimensões das vias aéreas.

**Objetivos:**

Avaliar as alterações em longo prazo nas dimensões das vias aéreas faríngeas em pacientes com Classe II esquelética tratados com aparelho extrabucal de puxada cervical.

**Material e Métodos:**

Foi realizado um estudo retrospectivo com 20 pacientes atendidos por um único ortodontista experiente. O espaço aéreo foi avaliado por meio de medidas lineares e de área em radiografias cefalométricas pré-tratamento, pós-tratamento e em pós-contenção de 25 anos.

**Resultados:**

As medidas apresentaram concordância elevada a moderada. Nenhuma das medidas apresentou diminuição após o tratamento. As áreas nasofaríngea, orofaríngea e hipofaríngea não foram modificadas após a intervenção com o aparelho extrabucal e mostraram uma tendência a estreitar ao longo do período de acompanhamento pós-tratamento. Em relação às medidas lineares, as dimensões nasofaríngeas permaneceram inalteradas ou aumentaram após o tratamento, retornando às dimensões iniciais na avaliação de seguimento.

**Conclusão:**

O tratamento ortopédico com aparelhos extraorais não provocou diminuição da dimensão das vias aéreas faríngeas e é considerado seguro para o tratamento de pacientes em crescimento com má oclusão esquelética de Classe II, de acordo com os resultados do presente estudo.

## INTRODUCTION

Nasopharyngeal obstruction is a serious condition that can impact on facial growth, cause malocclusion, and affect quality of life. It is also associated with obstructive sleep apnea (OSA) in children.^1^ ^,^ ^2^ This underscores the importance of understanding the role of orthodontic and orthopedic approaches in improving breathing. 

Despite the lack of consistent information, current evidence suggests that mandibular advancement therapies might benefit airway dimensions in the short term.^3^ ^,^ ^4^ On the other hand, the potential of rapid palatal expansion (RPE) to improve nasal airway obstruction depends on the pathological condition; being ineffective in patients with obstructive adenoids.^5^ Furthermore, scientific literature does not support the notion that orthodontic treatment with premolar extraction reduces pharyngeal airway volume. Instead, it suggests that mandibular anatomy and the degree of counterclockwise rotation after treatment may influence changes in airway dimensions due to orthodontic extraction therapy.^6^

Although maxillary expansion, premolar extraction, and mandibular protraction have been investigated,^3^ ^-^ ^8^ the role of cervical headgear (CHG) in changing pharyngeal dimensions remains less known.^9^ ^-^ ^11^ CHG is an established treatment for Class II, division 1 growing patients. This orthopedic appliance’s outcomes are maxillary widening and restriction of anterior displacement,^12^ ^,^ ^13^ which is associated with normal mandibular growth.^13^ ^,^ ^14^ These effects may impact upper airway space dimensions or modify respiratory air patency.

It has been suggested that CHG treatment could predispose to upper airway obstruction during sleep.^15^ ^,^ ^16^ In contrast, recent evidence indicates that there is no substantial negative effect on sleep attributes during orthodontic treatment with CHG.^17^ Although CHG treatment has been associated with an increase in the retropalatal airway space,^14^ no study has assessed its impact on airway dimensions in the long term.

This study aims to assess the long-term changes in pharyngeal airway dimensions of skeletal Class II patients treated with cervical headgear, followed by comprehensive fixed appliance therapy without extractions.

## MATERIAL AND METHODS

A retrospective study was conducted in accordance with the Ethics Code of the World Medical Association (Declaration of Helsinki) and the Ethics Board of the Ministry of Health (Resolution CNS/MS 466/2012) for research involving humans. The study received the ethics committee approval (#62558616.5.0000.5024).

The sample comprised 20 patients (14 females and 6 males), with a mean age of 12 years and 6 months. Participants, who had no recorded information concerning OSA symptoms, were treated by the same experienced clinician following the same protocol. All patients provided written informed consent to participate. 

Inclusion criteria were initial diagnosis of Angle Class II, division 1 malocclusion with a bilateral full Class II molar relationship; ANB > 4,5^o^, vertical skeletal pattern within a normal range (FMA=25°± 5); active growth potential, below the pubertal growth peak, and no growth-related diseases or craniofacial anomalies. Subjects with any tooth loss or major dental rehabilitation during the retention period were excluded.

The treatment approach involved cervical pull headgear with a force of 500 g/12 h/day, combined with an Edgewise standard fixed appliance without premolars extractions or intermaxillary elastics mechanics. Patients wore the CHG device until the right and left molar relationships were fully corrected, even if the full fixed appliance had already been initiated.

The mean period of headgear treatment was 2 years and 1 month, and the mean period of fixed appliance use was 3 years and 3 months, with an average total treatment time of 4 years and 4 months.

Lateral cephalograms obtained before treatment (T1); post-treatment (T2) and post-retention (T3), at least 25 years after treatment, were used for pharyngeal dimensions assessment.

Measurements were performed by a single expert operator using Dolphin Imaging 11.7 Premium software (Dolphin Imaging & Management Solutions, Chatsworth, CA, USA) and the SketchAndCalc software (SketchAndCalc^TM^ Area Calculation software, iCalc Inc., FL, USA) for area measurements. Image magnification was calibrated using the radiopaque ruler present in each radiography.

**Figure 1:**
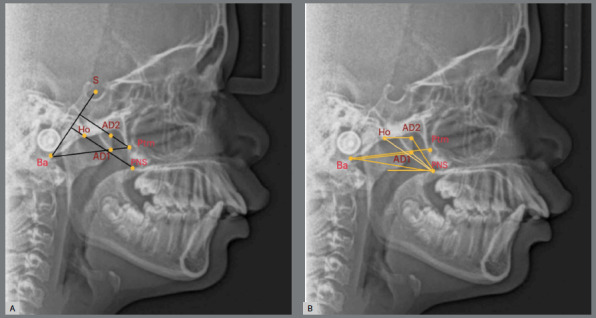
A) Nasopharyngeal airway landmarks: S (Sella); Ba (Basion); PNS (Posterior nasal spine); Ho (Hormion: located at the intersection between the perpendicular line to S-Ba from PNS and the cranial base); Ptm: (Pterygomaxillary fissure: most inferior point of pterygomaxillary fissure); AD1 (point of intersection of posterior pharyngeal wall [PPW] and line Ptm-Ba) and AD2 (intersection point of PPW and line from Ptm as normal perpendicular to S-Ba). B) Nasopharyngeal airway measures: AD1-PNS; AD1-Ba; AD2-PNS; AD2-Ho; PNS-Ba; Ptm-Ba; PNS-Ho and PPS (palatal pharyngeal distance: on the line passing from PNS-PPW, parallel to FH).

The sample was reevaluated under the same conditions by the same operator one month apart to ensure intra-examiner reliability of the method. The pharyngeal airway dimensions were assessed through radiographic cephalometric measurements, as previously described.^18^ A total of 18 cephalometric measurements were taken, with landmarks and measurements illustrated in Figures 1-4.

**Figure 2: f2:**
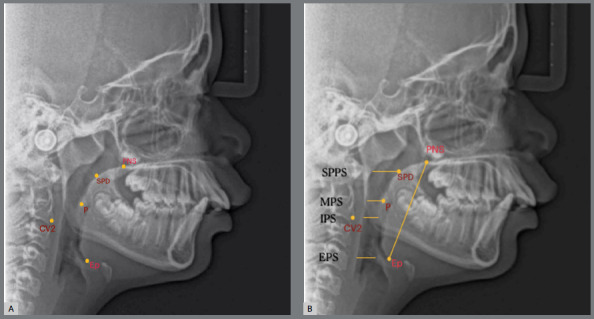
A) Oropharyngeal airway landmarks: PNS; SPD (soft palate dorsum; the midpoint of the soft palate); P (extreme point of the soft palate); Ep (epiglottis base, also the most posterior and inferior point in the tongue base) and CV2 (most inferior and anterior point of the second cervical vertebra). B) Oropharyngeal airway measures: PNS-Ep; SPPS (superior posterior pharyngeal space: on the line from SPD parallel to FH); MPS (middle pharyngeal space: on the line passing from P, parallel to FH); IPS (inferior pharyngeal distance: on the line passing from CV2, parallel to FH) and EPS (epiglottic pharyngeal distance: on the line passing from Ep, parallel to FH).

**Figure 3: f3:**
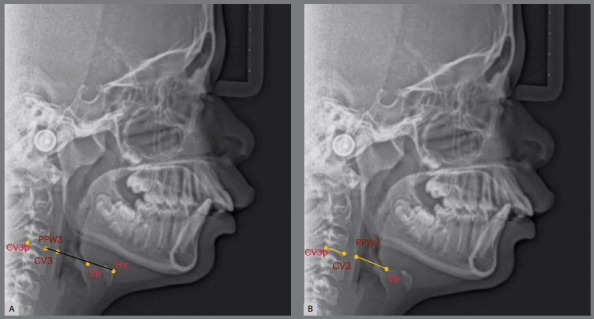
A) Hypopharyngeal airway landmarks: CV3p (most inferior and posterior point of the third cervical vertebra); CV3 (most inferior and anterior point of the third cervical vertebra); Hy (most superior and anterior point of hyoid bone); PPW3 (posterior pharyngeal wall along the line intersecting CV3 and Hy) and Ep. B) Hypopharyngeal airway measure: Ep-PPW3.

**Figure 4:**
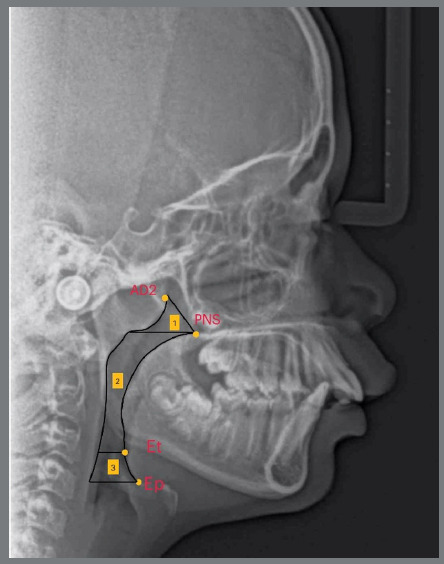
Area Measurements: (1) Nasopharynx; (2) Oropharynx and (3) Hypopharynx. Cephalometric landmarks: AD2; PNS; Et (end point of the epiglottis) and Ep.

## STATISTICAL ANALYSIS

Descriptive analysis was performed to identify the specific and general features of the sample. The average and standard deviation (SD) were calculated using the R software (4.4.0 version). 

To evaluate intra-rater agreement, the intraclass correlation coefficient (ICC) with a 95% confidence interval was used. To investigate statistically significant differences between cephalometric measurements over time, analysis of variance (ANOVA) for repeated measures was employed, followed by the Bonferroni test for multiple comparisons. A significance level of 5% was adopted.

## RESULTS

The measurements performed had a high or moderate intra-rater agreement, except for the nasopharyngeal area (Table 1).

**Table 1:** Intra-rater concordance for each cephalometric measurement.

Cephalometric measurements	Intraclass correlation coefficient (ICC)	Confidence Interval 95%
Linear measurements (mm)		
AD1-PNS	0.791	[0.329 - 0.932]
AD1-Ba	0.848	[0.532 - 0.949]
AD2-PNS	0.796	[0.379 - 0.932]
AD2-Ho	0.893	[0.692 - 0.964]
PNS-Ba	0.943	[0.834 - 0.981]
Ptm-Ba	0.844	[0.544 - 0.947]
PNS-Ho	0.901	[0.697 - 0.967]
PPS	0.783	[0.255 - 0.931]
PNS-Ep	0.979	[0.939 - 0.993]
SPPS	0.896	[0.700 - 0.965]
MPS	0.795	[0.418 - 0.930]
IPS	0.943	[0.836 - 0.981]
EPS	0.947	[0.844 - 0.982]
EpPPW3	0.963	[0.894 - 0.988]
Area measurements (mm^2^)		
Nasopharyngeal space	0.376	[-0.976 - 0.794]
Oropharyngeal space	0.887	[0.673 - 0.962]
Hypopharyngeal space	0.909	[0.738 - 0.969]
Total pharyngeal area	0.929	[0.786 - 0.976]

Font: Research Data.

The descriptive statistics for all measurements are presented in Table 2. There was no statistically significant difference in airway dimensions between T1 and T2 for AD1-PNS, Ptm-Ba, PNS-Ho, PPS, SPPS, and MPS. However, at the long-term follow-up (T3), these measurements showed a statistically significant decrease compared to T1 and T2.

**Table 2:** Descriptive statistics in T1, T2 and T3.

Cephalometric Measurements (mm)	T1	T2	T3
	Average	Average	Average
	(standard deviation)	(standard deviation)	(standard deviation)
AD1-PNS	26.3 (2.8)^a^	26.4 (2.9)^a^	22.2 (3.2)^b^
AD1-Ba	20.5 (2.4)^a^	21.1 (3.7)^a^	22.4 (4.9)^a^
AD2-PNS	19.0(3.0)^a^	20.7 (2.6)^b^	17.9 (4.3)^a^
AD2-Ho	11.1 (3.4)^a^	10.1 (3.1)^a^	9.5 (3.9)^a^
PNS-Ba	46.8 (3.0)^a^	47.4 (4.1)^ab^	44.6 (5.0)^ac^
Ptm-Ba	45.6 (3.4)^a^	46.2 (3.3)^a^	43.9 (3.2)^b^
PNS-Ho	29.9 (2.6)^a^	30.8 (3.5)^a^	27.5 (4.3)^b^
PPS	27.0 (3.0)^a^	26.9 (2.9)^a^	23.1 (2.8)^b^
PNS-Ep	60.7 (5.5)^a^	65.5 (7.3)^b^	61.6 (7.3)^a^
SPPS	13.3 (3.5)^a^	12.7 (3.8)^a^	10.6 (2.9)^b^
MPS	10.2 (2.5)^a^	10.3 (2.6)^a^	8.5 (2.2)^b^
IPS	10.8 (4.2)^a^	11.7 (3.9)^ab^	9.0 (3.3) ac
EPS	9.10 (3.3)^a^	10.4 (3.2)^ab^	7.7 (2.9) ac
Ep-PPW3	26.6 (3.5)^a^	30.1 (3.8)^b^	26.6 (3.4)^a^
Área nasofaríngea	127.0 (34.0)^a^	143.0 (42.0)^a^	130.0 (39.0)^a^
Área orofaríngea	466.0 (129)^a^	519.0 (113)^ab^	442.0 (92.0)^ac^
Área hipofaríngea	103.0 (32.0)^a^	124.0 (43.0)^a^	101.0 (36.0)^a^
Total pharyngeal area	697.2 (167)^a^	787.0 (162)^b^	674.0 (138)^a^

Similar Letters indicate no statistical difference. Font: Research Data.

The measurements AD1-Ba; AD2-Ho; nasopharyngeal area and hypopharyngeal area remained stable and did not show statistical differences over the assessed periods.

Some linear measurements, such as AD2-PNS, PNS-Ep, Ep-PPW3, and total airspace area, increased from T1 to T2 but did not maintain this increase at the follow-up (T3), showing no statistical difference between T1 and T3.

Lastly, the measurements PNS-Ba; IPS; EPS, and oropharyngeal area exhibited statistical differences only between T2 and T3, with a slight decrease. However, this decrease was not statistically significant when compared to the initial observation.

## DISCUSSION

As a classic appliance for correcting class II malocclusion in growing patients, cervical headgear (CHG) has been extensively studied, with well-documented effects on facial features, occlusion, and skeletal development.^12^ ^,^ ^13^ ^,^ ^19^ However, there are few studies in the literature^9^ ^-^ ^11^ assessing the impact of this treatment in the airway characteristics; the results are conflicting and only one recent study evaluated the airway dimensions with CHG treatment over time.^10^ To the best of our knowledge, no study has assessed the upper airway dimensions in patients treated with headgear by a single experienced orthodontist with such a long-term follow-up. This information is critical, as airway constrictions are associated with serious breathing disorders that have systemic and social impacts. The concern in orthodontics about obstructive sleep apnea (OSA) has increased significantly, prompting numerous studies to clarify the consequences of different approaches on airway dimensions.^3^ ^-^ ^8^ ^,^ ^20^

Although the use of CHG has decreased over time, with fixed functional devices gaining popularity due to their aesthetics and reduced dependence on patient compliance,^21^ headgear remains in clinical use. It is considered the most effective orthopedic treatment for maxillary protrusion,^13^ does not cause proclination of lower incisors, and allows adjustments to accommodate different vertical patterns.^12^ ^,^ ^19^ Therefore, evaluating the long-term outcomes of this traditional device is essential to justify its continued use as an orthopedic treatment option.

The results of this study showed that none of the airway dimensions decreased after headgear treatment, what is in accordance with a recent research.^10^ The nasopharynges, oropharynges, and hypopharynx remained unchanged post-treatment, although there was a tendency for these dimensions to widen slightly with the intervention, without statistical significance. Otherwise, in the long-term follow-up, the airway space tended to narrow, which could be attributed to factors such as weight gain and decreased muscle tone over the years, affecting the thickness of the airway space.

Interpretation of these results must consider the study’s limitations, such as the absence of a control group of untreated skeletal Class II patients. Even though an untreated Class II sample is available for use, proper comparison would not be possible due to ethnic background differences. This is a common limitation in health research, as it is unethical to leave a group of patients untreated. Additionally, non-probability sampling presents bias and power issues that are difficult to measure. However, convenience sampling in retrospective long-term research in health sciences is often justified by research feasibility, availability, and cost-effectiveness.

Cephalometric radiography, widely used in orthodontics, is a low-cost, low-radiation exam that provides valuable information for diagnosis and planning. It is an efficient^22^ and reliable^23^ method for evaluating nasopharyngeal and oropharyngeal dimensions and is validated for scientific research.^24^ In the present study, a reliable information could be extracted without exposing patients to unnecessary radiation, such as the tomographic exam. Moreover, we recommend that orthodontists include morphometric assessment of patient’s airway features in their standard cephalometric analysis.

This study focused on normodivergent patients, although vertical patients are at a higher risk of respiratory impairments,^25^ ^,26^ and airway stenosis. ^27^ Thus, future research should investigate the effect of high-pull headgear treatment on airway dimensions.

The effectiveness^19^ and long-term stability of skeletal Class II correction with headgear are well-documented.^12^ This classic appliance, with a history of nearly 80 years, remains relevant even as the field evolves with new technologies and approaches for treating skeletal Class II malocclusions. It is important to determine whether headgear should be phased out or continue to play a role in orthopedic treatment, given its low cost, high efficiency, lack of negative impact on lower incisors and airway dimensions, and ease of use during sleep, despite its reliance on patient compliance.

## CONCLUSION

There is no negative effect on upper airway dimensions with cervical headgear orthopedic treatment in the long term. This new information may encourage orthodontists to continue using this appliance in their clinical arsenal to treat growing skeletal Class II patients.
